# Gentiopicroside, a Secoiridoid Glycoside from *Gentiana rigescens* Franch, Extends the Lifespan of Yeast via Inducing Mitophagy and Antioxidative Stress

**DOI:** 10.1155/2020/9125752

**Published:** 2020-08-02

**Authors:** Qian Liu, Lihong Cheng, Akira Matsuura, Lan Xiang, Jianhua Qi

**Affiliations:** ^1^College of Pharmaceutical Sciences, Zhejiang University, Yu Hang Tang Road 866, Hangzhou 310058, China; ^2^Department of Biology, Graduate School of Science, Chiba University, Chiba 263-8522, Japan

## Abstract

Gentiopicroside (GPS), an antiaging secoiridoid glycoside, was isolated from *Gentiana rigescens* Franch, a traditional Chinese medicine. It prolonged the replicative and chronological lifespans of yeast. Autophagy, especially mitophagy, and antioxidative stress were examined to clarify the mechanism of action of this compound. The free green fluorescent protein (GFP) signal from the cleavage of GFP-Atg8 and the colocation signal of MitoTracker Red CMXRos and GFP were increased upon the treatment of GPS. The free GFP in the cytoplasm and free GFP and ubiquitin of mitochondria were significantly increased at the protein levels in the GPS-treated group. GPS increased the expression of an essential autophagy gene, *ATG32* gene, but failed to extend the replicative and chronological lifespans of *ATG32* yeast mutants. GPS increased the survival rate of yeast under oxidative stress condition; enhanced the activities of catalase, superoxide dismutase, and glutathione peroxidase; and decreased the levels of reactive oxygen species and malondialdehyde. The replicative lifespans of *Δsod1*, *Δsod2*, *Δuth1*, and *Δskn7* were not affected by GPS. These results indicated that autophagy, especially mitophagy, and antioxidative stress are involved in the antiaging effect of GPS.

## 1. Introduction

Aging is a time-dependent functional decline that is the primary risk factor for many diseases [1, 2], such as Alzheimer's disease, Parkinson's disease, and diabetes. With the intensive increase in the aging population worldwide, enormous personal, social, and economic challenges have been encountered by the modern society [3]. Therefore, studies on prolonging the lifespan, especially improving the healthspan to increase the quality of life and retard suffering of inevitability of elders, have been intensively conducted worldwide [4].

*Gentiana rigescens* (*G*. *rigescens*) grows in the southwest region of China. The roots of this Chinese herb medicine have been used to treat inflammation, hepatitis, and functional dyspepsia [5]. *Sheng Nong's Herbal Classic*, a classic book of traditional Chinese medicine on Materia Medica, described that *G*. *rigescens* has effects on cognition improvement and antiaging. In our previous study, 11 novel neuritogenic benzoate-type molecules (named gentisides A–K) were isolated from *G*. *rigescens* [6, 7]. The mixture of gentisides (n-GS) was confirmed to have alleviation effects on the impaired memory of scopolamine-induced mouse model by inhibiting acetylcholinesterase activities and antioxidative stress and regulating the insulin-like growth factor 1 receptor/extracellular signal-regulated kinase (IGF-1R/ERK) signal pathway [8]. These results possibly proved the cognition-improving effect of *G*. *rigescens* on the molecular basis, which was mentioned in *Sheng Nong's Herbal Classic*.

In the present study, we investigated the antiaging molecular base of *G*. *rigescens*. Budding yeast *Saccharomyces cerevisiae* is a common aging model that has replicative and chronological lifespans. The K6001 strain, a yeast mutant strain derived from W303, has a characteristic that only mother cells can produce daughter cells and daughter cells do not continue to mitosis in glucose medium [2, 9]. The use of K6001 as a model to study replicative lifespan possesses many merits, such as low cost, short generation time, and easy operation. Therefore, the replicative lifespan assay of K6001 was utilised as a bioassay system to screen antiaging substances from food extracts and traditional Chinese medicines.

Autophagy is a highly conservative process used to degrade large structures, such as long-lived or damaged organelles and protein aggregates [10]. Accordingly, autophagy serves an adaptive role to protect organisms against diverse pathologies, including infections, cancer, neurodegeneration, aging, and heart disease [11]. The regulation of autophagy is extremely important for the maintenance of normal physiological functions [12]. Reports show that enhancing autophagy can regulate aging and aging-related diseases and prolong lifespan [13]. For example, rapamycin, a well-known molecule, as an antiaging candidate, is reported to extend the lifespan of *Caenorhabditis elegans* (*C*. *elegans*), fruit fly, and mice, but the effects are abolished when the autophagy-related genes are knocked out or knocked down, suggesting that autophagy plays an important role in the life-prolonging effect of rapamycin [14, 15]. Mitophagy, a selective type of autophagy, which targets long-lived or damaged mitochondria to degradation, is regarded as a major mechanism responsible for mitochondrial quality control. The accumulation of damaged mitochondria is a common marker of aging and is related to diseases. However, the level of mitophagy markedly declines in mammalian tissues during normal aging, and the decline in mitophagy might fuel the vicious circle of induced oxidative stress and age-related tissue damage [16]. Many studies have shown that stimulating mitophagy might have widespread beneficial effects in antiaging or age-related functional decline. For example, urolithin A, a metabolite of natural products, extends the lifespan of *C*. *elegans*, depending on the expression levels of the mitophagy genes *pink-1*, *dct-1*, and *skn-1* and improves the muscle function in old mice with a high expression of mitophagy (*Park2*) transcripts [17].

Oxidative stress is a disturbance in the balance between the production of free radicals and antioxidant defences, thereby causing cell aging. Free radicals, known as reactive oxygen species (ROS) and reactive nitrogen species [18], will be excessively produced when cells are under oxidative stress condition. The superfluous free radical attacks cell components and produces harmful substances, such as malondialdehyde (MDA), the product of lipid oxidation that can cause cross-linking polymerisation of proteins, nucleic acids, and other biological macromolecules [19]. However, these types of oxidative damage can be alleviated by antioxidant enzymes, such as superoxide dismutase (SOD), catalase (CAT), and glutathione peroxidase (GPx). In our previous published paper, *SOD1*, *SOD2*, SUN family protein Uth1 (*UTH1*), and *SKN7* genes were confirmed to be involved in oxidative stress and played an important role in the antiaging effects [20].

In our previous study, many antiaging substances were isolated from natural products, such as parishin, phloridzin, cucurbitacin B, cucurbitane glycosides, and a new compound named bis(4-hydroxybenzyl)ether mono-*β*-L-galactopyranoside from *Gastrodia elata*, apple branches, *Pedicellus melo*, fruits of *Momordica charantia* L and *G*. *elata* Blume, under the guidance of the K6001 yeast bioassay system [2, 20–23]. In the present study, GPS was isolated from *G*. *rigescens* using the same bioassay system. Here, the antiaging activity and the mechanism of action of GPS were reported.

## 2. Materials and Methods

### 2.1. General

*G*. *rigescens* was purchased from Huqingyutang Chinese Pharmacy Hangzhou, Zhejiang Province. The identity of this plant was confirmed by Associate Professor Liurong Chen (College of Pharmaceutical Sciences, Zhejiang University), and a voucher specimen (no. 20190620) was preserved in Zhejiang University, Institute of Materia Medica. Preparative high-performance liquid chromatography (HPLC) analysis was performed using a HPLC system equipped with Elite P-230 pumps (Dalian Elite Inc., China). An Agilent Technologies 6224A Accurate-Mass time-of-flight liquid chromatography–mass spectrometry (TOF LC/MS) system was used to measure the mass spectra (Agilent Technologies Inc., Beijing, China). A Bruker AV III-500 spectrometer was used to record the nuclear magnetic resonance (NMR) spectra (Bruker, Karlsruhe, Germany). NMR chemical shifts in *δ* (ppm) were referenced to solvent peaks of *δ*_C_ 49.0 for CD_3_OD (Cambridge Isotope Laboratories, Inc., MA, USA). Octadecyl-silane (Cosmosil 75 C18-OPN, Nacalai Tesque, Japan) and RP-18 plates (0.25 mm) (Merck KGaA, Darmstadt, Germany) were used for column chromatography and thin-layer chromatography analysis, respectively [2]. Resveratrol (Res) was purchased from J&K Scientific Ltd. (Beijing, China) and was used as a positive control.

### 2.2. Preparation and Determination of GPS

**Figure 1:**
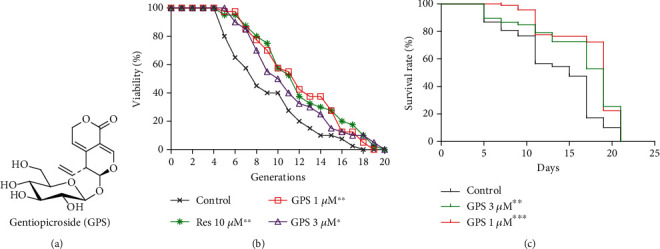
Chemical structure and effect of GPS on the lifespan of yeast. (a) Chemical structure of GPS. (b) The changes in the replicative lifespan of K6001 yeast after treatment of GPS. RES at 10 *μ*M is used as the positive control. (c) Effect of GPS on the chronological lifespan of YOM36 yeast. ^∗^,^∗∗^, and ^∗∗∗^ represent significant difference compared with the control group at *p* < 0.05, *p* < 0.01, and *p* < 0.001, respectively. The chronological lifespan assay is performed in the SC medium, and the survival rate less than 5% of each group is defaulted to 100% death.

GPS was isolated from *G*. *rigescens*. Dried and powdered *G*. *rigescens* (100 g) was extracted in methanol (material/methanol, 1/10, *w*/*v*) at room temperature for 24 h with shaking. The methanol extract was filtrated, and the filtrate was concentrated, suspended in water, and successively partitioned with *n*-hexane, ethyl acetate, and *n*-butanol to obtain the active *n*-butanol fraction (7.5 g). The n-butanol fraction was separated by ODS open column and eluted with MeOH/H_2_O (20 : 80, 30 : 70, 40 : 60, 50 : 50, 70 : 30, 90 : 10, and 100 : 0) to give seven fractions. A part of the third fraction (30 mg) was purified through HPLC (Cosmosil 5C30-UG-5 (10 mm × 250 mm), flow rate: 3 mL/min, 30% aq. MeOH) to yield a pure active substance (23 mg, *t*_R_ = 44 min). The chemical structure of the active molecule was identified to be gentiopicroside (GPS) by comparing HR ESI-MS and ^13^C NMR data with those reported in literature [24]. ^13^C NMR (125 MHz, CD_3_OD): *δ* = 166.3 (C-11), 150.6 (C-3), 135.0 (C-8), 127.0 (C-5), 118.5 (C-10), 117.2 (C-6), 105.0 (C-4), 100.2 (C-1′), 98.5 (C-1), 78.4 (C-5′), 78.0 (C-3′), 74.6 (C-2′), 71.5 (C-4′), 70.9 (C-7), 62.8 (C-6′), and 46.6 (C-9); and high-resolution ESI-TOF-MS *m*/*z* 379.099, calcd for C_16_H_20_NaO_9_ (M+Na)^+^379.100. The chemical structure of GPS is shown in Figure 1(a).

### 2.3. Yeast Strains and Lifespan Assay

The K6001 yeast, *Δuth1*, *Δskn7*, *Δsod1*, *Δsod2*, and *Δatg32* yeast mutants with K6001 background, YOM36, *Δatg32* of YOM36, YOM38-Atg8-GFP, and BY4741 yeast strains were used in the present study. K6001 yeast was provided by Professor Breitenbach (Salzburg University, Austria), and other mutant yeast strains were constructed by Professor Matsuura (Chiba University, Japan). The replicative lifespan assay was performed in accordance with the protocol described in our previous study [2, 20]. Briefly, the K6001 yeast was inoculated in galactose medium (3% galactose, 2% hipolypeptone, and 1% yeast extract) for 24 h in a shaking incubator at 180 rpm and 28°C. The harvested yeast cells were then washed with PBS for three times, and approximately 4000 cells were spread on glucose agar plates (2% glucose, 2% hipolypeptone, 1% yeast extract, and 2% agar) containing GPS at concentrations of 0, 0.3, 1, 3, and 10 *μ*M. The well-known antiaging molecule, Res, at a concentration of 10 *μ*M was used as positive control. The agar plates were then incubated at 28°C, and 40 microcolonies formed on agar plate were randomly selected and counted daughters produced by each mother cell under microscopy. The replicative lifespan assay methods of mutants with K6001 background (*Δuth1*, *Δskn7*, *Δsod1*, *Δsod2*, and *Δatg32* of K6001) were the same as that of the K6001 strain. The chronological lifespan assay was conducted as described in our previous report [2, 22]. YOM36 yeast were cultured in synthetic complete (SC) medium (2% glucose, 2% peptone, and 1% yeast extract) or synthetic defined (SD) medium (0.17% yeast nitrogen base without amino acids and ammonium sulphate (BD Difco), 0.5% ammonium sulphate, and 0.2% glucose) in a shaking incubator at 180 rpm and 28°C. After 24 h, the cells were inoculated into the SC or SD medium containing GPS at concentrations of 0, 1, and 3 *μ*M with the initial OD600 value of 0.01 and incubated in a shaking incubator at 180 rpm and 28°C. On the third day, 200 cells from each treat or untreated groups were spread on glucose agar plates and cultured in an incubator at 28°C for 2 days. The colony-forming units (CFUs) of each plate were counted, and the CFUs were denoted as 100% survival at day 3. On the fifth day, the steps of the third day were repeated, and the survival rate of each group was calculated (CFUs of each plate/CFUs of the same plate on the third day × 100%). The steps were repeated every two days until the survival rate decline to less than 10%. The chronological lifespan assay methods of YOM36 mutants (*Δatg32* of YOM36) were the same as those of YOM36 strain.

### 2.4. Visualisation of Autophagy and Mitophagy of Yeast through Confocal Fluorescence Microscopy

The experiment was performed following a previously published literature [22]. Briefly, YOM38 yeast cells containing pR316-GFP-Atg8 plasmid were cultured in liquid glucose medium in a shaking incubator at 180 rpm and 28°C under dark conditions. After 24 h, the cells were collected and washed with SD medium and divided into several different groups with OD600 value of 0.1. The cells were then treated with GPS at concentrations of 0, 1, and 3 *μ*M or Res at 300 *μ*M and cultured for 22 h in the dark. Subsequently, the cells were stained with 4′,6-diamidino-2-phenylindole (DAPI) 20 *μ*g/mL) for 10 min in the dark and then washed thrice with PBS. Yeast cells were observed and photographed with a two-photon confocal fluorescence microscope (Olympus FV1000BX-51, Tokyo, Japan). The experimental procedure for mitophagy was similar to that of autophagy. The difference is that the cells were stained with 250 nM MitoTracker Red CMXRos (Beyotime, Shanghai, P. R. China) at 37°C in the dark for 1 h before staining with DAPI. The percentage of cells with green fluorescence and the colocation of red and green fluorescence were calculated, and the data obtained were analysed on software.

### 2.5. Western Blot Analysis

YOM38 yeast cells containing pR316-GFP-Atg8 plasmid were cultured in liquid glucose medium in a shaking incubator at 180 rpm and 28°C under dark conditions. After 24 h, the cells were collected and washed with SD medium and divided into several different groups with OD600 value of 0.1. The cells were then treated with GPS at concentrations of 0, 1, and 3 *μ*M or Res at 300 *μ*M and cultured for 22 h in the dark. Subsequently, yeast cells of different groups were collected and sonicated for 5 min. The cell lysates were centrifuged at 12,000 g for 15 min, and the supernatant was obtained for western blot analysis. The mitochondria were isolated from yeasts to obtain the protein, as described in references [25, 26]. Briefly, YOM38 yeast cells containing pR316-GFP-ATG8 plasmid were treated GPS similar to the above description. Subsequently, the cell lysate was centrifuged twice for 5 min at 5000 g. The supernatants were removed to new tubes and centrifuged for 15 min at 12,000 g to obtain the mitochondrial pellet. The mitochondrial pellet was dissolved in a RAPI lysis buffer and incubated in ice for 20 min. The supernatants were obtained as protein sample after centrifugation at 12,000 g for 15 min. The protein concentrations were measured with a BCA Protein Assay Kit (CoWin Biotech, Beijing, China). Western blot analysis was performed as described in our previous study [22]. Approximately 20 *μ*g protein was separated with SDS-PAGE and transferred to PVDF membranes. The membranes were incubated with primary antibodies followed by secondary antibodies. The primary antibodies used are as follows: anti-GFP (Medical & Biological Laboratories, Nagoya, Japan), anti-*β*-actin (CoWin Biotech, Beijing, China), antiubiquitin (Cell Signalling Technology, Massachusetts, USA), and anti-VDAC1/Porin (Abcam Trading (Shanghai) Company Ltd., Shanghai, China) antibodies. The secondary antibodies used are as follows: horseradish peroxidase-linked antirabbit and antimouse IgGs (CoWin Biotech, Beijing, China). Antigens were visualised using an ECL Western Blot Kit (CoWin Biotech, Beijing, China) and digitised with ImageJ software.

### 2.6. Real-Time Polymerase Chain Reaction (RT-PCR) Analysis

The wild-type BY4741 were incubated with GPS at concentrations of 0 and 1 *μ*M in glucose medium for 12 h at 28°C with shaking at 180 rpm. Total RNA was extracted using a hot phenol method. A reverse transcription method was utilised to synthesise cDNA using a HiFi-MMLV cDNA Kit (CoWin Biotech, Beijing, China) and 5 *μ*g of RNA. Quantitative RT-PCR was performed by using CFX96 Touch (Bio-Rad, Hercules, USA) and SYBR Premix EX Taq (Takara, Otsu, Japan), as described in our previous study [2, 20]. The thermal cycling parameters are as follows: 40 cycles, 94°C for 15 s, 51.6°C for 15 s, and 68°C for 20 s. The sequences of the primers for RT-PCR are as follows: for *ATG32*, sense 5′-ACC GTC TCA TCC CTT TAA AC-3′ and antisense 5′-CTT CCT CAA AAG CCT CAT CT-3′ and for TUB1, sense 5′-CCA AGG GCT ATT TAC GTG GA-3′ and antisense 5′-GGT GTA ATG GCC TCT TGC AT-3′. The relative gene expression data were analysed using the 2^−*ΔΔ*Ct^ method. The levels of *ATG32* mRNA were normalised to those of *TUB1.*

### 2.7. Antioxidative Assay

BY4741 yeast cells in glucose medium with initial OD600 value of 0.1 were treated with GPS at doses of 0, 1, and 3 *μ*M or Res as positive control at 10 *μ*M for 24 h. Subsequently, the yeast cells from each group were diluted to 1.5 OD600 value. Approximately 5 *μ*L of yeast culture from each group was dropped on glucose agar plates containing H_2_O_2_ at a dose of 10.5 mM and incubated at 28°C for 3 days. The growth of yeast was observed and photographed.

To quantify the antioxidative activity of GPS, BY4741 yeast cells were cultured with GPS at concentrations of 0, 1 and 3 *μ*M or 10 *μ*M Res at 28°C for 24 h. Then, 200 yeast cells from each treated group were painted on glucose agar plates with or without 5 mM of H_2_O_2_. After 2 days, the growth of yeast was observed, and the number of microcolonies in each plate was counted to evaluate the antioxidative activity. The survival rate was calculated as the ratio of the number of microcolonies with H_2_O_2_ at 5 mM divided by the number of microcolonies in the absence of H_2_O_2_.

### 2.8. CAT, GPx, and SOD Enzyme Activity Assays

BY4741 yeast cells were cultured in liquid glucose medium to reach the logarithmic growth phase. The cells were divided into five groups with OD600 value of 0.1 and treated with GPS at 0, 1, 3, and 10 *μ*M or Res at 10 *μ*M and cultured in a glucose medium for 24 h. Subsequently, the yeast cells from different groups were collected and sonicated for 5 min. The cell lysates were centrifuged at 12,000 g for 15 min, and the supernatant was obtained to test the activity of enzymes. The activities of CAT, GPx, and SOD were determined with CAT and GPx assay kits (Beyotime Biotechnology Limited Company, Shanghai, China) and SOD assay kits (Nanjing Jiancheng Bioengineering Institute (Nanjing, China) in accordance with the manufacturer's instructions. All experimental procedures were conducted in strict accordance with the protocol instructions provided by the manufacturers.

### 2.9. Measurement of ROS and MDA Level in Yeast

ROS and MDA assays were performed by following the same methods as reported in literature [20]. Briefly, BY4741 yeast cells were treated with GPS at 0, 1, 3, and 10 *μ*M or Res at 10 *μ*M and cultured for 24 h. Subsequently, DCFH-DA (2′,7′-dichlorodihydrofluorescein diacetate) was added to the culture to obtain a final concentration of 40 *μ*M. The cells were then incubated in a shaker under the dark condition at 28°C for 1 h. The cells were then collected and washed with PBS thrice. The DCF fluorescence intensity of 1 × 10^7^ cells were detected with a fluorescence plate reader using 488 nm as excitation and 525 nm as emission wavelengths.

To detect the MDA level in yeast, BY4741 yeast cells were cultured as described in the ROS assay for 24 h. These cells were then washed with PBS thrice, suspended in 250 *μ*L PBS, and ultrasonicated on ice for 5 min. Subsequently, these cells were centrifuged at 4°C for 10 min at 12,000 g to obtain the supernatant, and the MDA level was measured with a MDA assay kit (Nanjing Jiancheng Bioengineering Institute, Nanjing, China) in accordance with the manufacturer's instruction.

### 2.10. Statistical Analysis

Experimental data were expressed as mean ± SEM value of two or three independent experiments. Significant differences among the groups in all experiments were analysed through one-way ANOVA, followed by two-tailed multiple *t*-tests with Bonferroni's correction on GraphPad Prism 5 (GraphPad Software Inc.). Survival analysis was used for chronological lifespan assay. A *p* value of less than 0.05 was considered statistically significant.

## 3. Results

### 3.1. GPS Extends the Replicative and Chorological Lifespans of Yeast

Yeast as an aging model is simple and amenable to genetic and molecular manipulations [27]. It is widely used to study aging mechanisms and screen potential drug candidates for attenuating aging. In this study, a K6001 yeast replicative lifespan bioassay system was used as a bioassay system to screen antiaging samples. Res was used as a positive control to evaluate the reliability of the yeast bioassay system and bioassay results. The antiaging potential of GPS at concentrations of 0, 0.3, 1, 3, 10, and 30 *μ*M was evaluated using the K6001 replicative lifespan assay (Figure 1(b) and S1). The average replicative lifespan of each treatment or negative control group is as follows: 8.05 ± 0.60 generations for the negative control group, 11.13 ± 0.67 generations for the positive control group treated with Res at 10 *μ*M, 8.08 ± 0.51 generations for treatment with GPS at 0.3 *μ*M, 11.35 ± 0.64 generations for treatment with GPS at 1 *μ*M, 10.25 ± 0.63 generations for treatment with GPS at 3 *μ*M, 10.48 ± 0.73 generations for treatment with GPS at 10 *μ*M, and 9.73 ± 0.59 generations for treatment with GPS at 30 *μ*M. These results indicated that GPS can significantly prolong the replicative lifespan of K6001 yeast cells at concentrations of 1, 3, and 10 *μ*M (*p* < 0.01, *p* < 0.05, and *p* < 0.05), respectively. The chronological lifespan assay of YOM36 yeast was performed to evaluate the antiaging activity of GPS. GPS significantly increased the survival rate of yeast at concentrations of 1 and 3 *μ*M (*p* < 0.001, *p* < 0.01) (Figure 1(c)). These results revealed that GPS extends the replicative and chronological lifespans of yeast.

### 3.2. Effects of GPS on Autophagy and Mitophagy in Yeast

**Figure 2:**
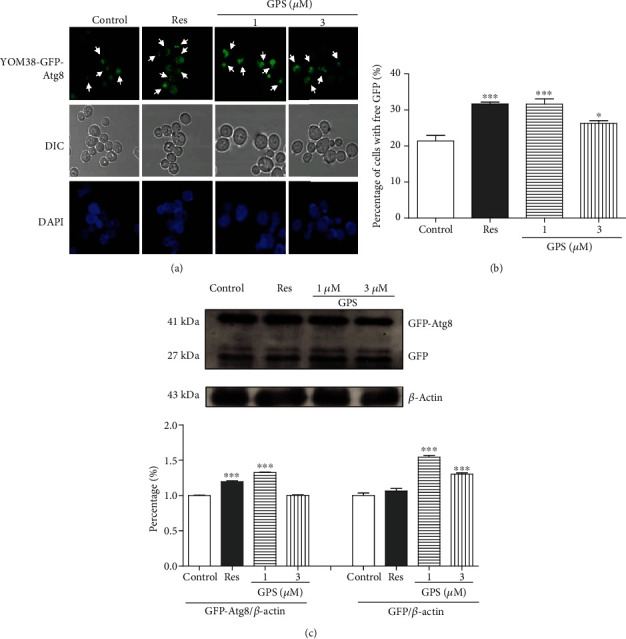
Effect of GPS on autophagy in yeast. (a) Fluorescent images of YOM38 yeast containing plasmid pR316-GFP-Atg8 after treatment of RES or GPS observed with a two-photon confocal fluorescent microscope. (b) The percentage of YOM38 cells containing plasmid pR316-GFP-Atg8 with autophagosome (green). Three pictures containing more than 60 cells in each group are used for statistical analysis. (c) Western blot analysis of GFP-Atg8 and free GFP in yeast after treatment with RES or GPS for 22 h in the SD medium. ^∗^ and ^∗∗∗^ indicate significant differences from the corresponding control at *p* < 0.05 and *p* < 0.001, respectively. Each experiment is repeated twice.

Autophagy, particularly mitophagy, is a degenerative process that degrades cellular components, such as the mitochondria, for recycling into amino acids and other metabolites. Autophagy and aging is closely related [13, 28]. Therefore, the effects of GPS on autophagy and mitophagy were examined. We used the YOM38-GFP-Atg8 yeast strain, which expresses GFP-Atg8 at a physiological level, to monitor the level of free GFP upon the treatment with GPS through fluorescent microscopy. The fluorescent images are displayed in Figure 2(a), and the digital result is shown in Figure 2(b). GPS significantly enhanced the percentage of cells with free GFP from 21.4 ± 1.6% to 31.6 ± 1.5% and 26.23 ± 0.8% at the concentrations of 1 and 3 *μ*M (*p* < 0.001 and *p* < 0.05), respectively. The positive control Res at 300 *μ*M increased the percentage from 21.4 ± 1.6% to 31.7 ± 0.5% (*p* < 0.001). To confirm the effect of GPS on the regulation of autophagy, western blot analysis was performed to test the generation of free GFP that is released into the vacuole during the autophagy flux. The results of western blot analysis are displayed in Figure 2(c) and Figure S2. GPS significantly increased the expression levels of Atg8-GFP and free GFP protein at the concentration of 1 *μ*M (*p* < 0.001). These results implied that GPS can increase the level of autophagy in yeast.

**Figure 3:**
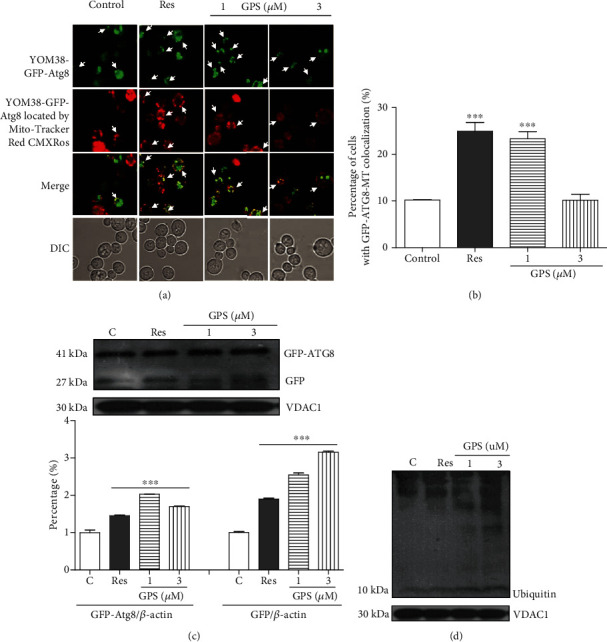
Effect of GPS on mitophagy in yeast. (a) Fluorescent images of YOM38 yeast containing plasmid pR316-GFP-Atg8 after treatment of GPS through MitoTracker Red CMXRos staining. (b) The percentage of YOM38 cells containing plasmid pR316-GFP-Atg8 with the colocation of autophagosome (green) and MitoTracker Red CMXRos (red). (c, d) The changes in ATG8-GFP, free GFP, and ubiquitin at the protein level in the mitochondria after treatment of GPS. Three pictures containing more than 60 cells in each group are used for statistical analysis.

Mitochondria are the sites of oxidative phosphorylation and ROS production, such as H_2_O_2_ and superoxide anion (O_2_^·−^) in eukaryotic cells [29]. Hence, mitophagy, a selective type of autophagy, is extremely important in degrading the damaged mitochondria. MitoTracker Red CMXRos to localise the mitochondria of YOM38-GFP-Atg8 cells (Figure 3(a)) was used to monitor mitophagy. GPS increased the percentage of cells with the colocation of green and red fluorescence from 10.2 ± 0.1% to 23.3 ± 1.5% at the concentrations of 1 *μ*M (Figure 3(b)) (*p* < 0.001). GPS increased the Atg8-GFP and free GFP protein levels in the mitochondrial fraction of yeast (Figure 3(c) and Figure S2, *p* < 0.001). The ubiquitin-proteasome system is a major intracellular pathway for the degradation of proteins that govern important life processes of the cell [17]. Therefore, we tested the expression of ubiquitin in the mitochondria of yeast at the protein level and found that GPS can enhance the expression of ubiquitin at the protein level (Figure 3(d) and Figure S2). These results revealed that GPS can significantly induce authophagy and mitophagy in yeast.

### 3.3. ATG32 Involved in the Antiaging Effect of GPS

**Figure 4:**
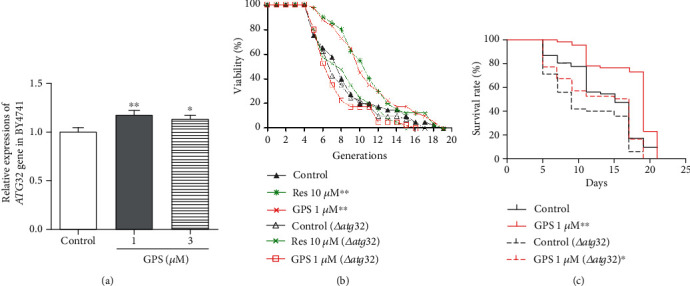
Effect of GPS on *ATG32* gene expression and lifespan of *Δatg32* mutant yeast with K6001 and YOM36 backgrounds. (a) Effect of GPS on *ATG32* gene expression after 12 h treatment. (b) Effect of GPS on the replicative lifespan of *atg32* mutants with K6001 background. (c) Effect of GPS on the chronological lifespan of *atg32* mutants with YOM36 background. ^∗^, ^∗∗^, and ^∗∗∗^ indicate significant differences from the corresponding control at *p* < 0.05, *p* < 0.01, and *p* < 0.001, respectively. Each experiment is repeated twice.

In yeast, the Atg32 protein localises on the mitochondria, interacts with Atg8 and Atg11, and plays an important role on mitophagy [30]. Therefore, we explored the effects of GPS on the *ATG32* gene expression in yeast (Figure 4(a)). The PCR analysis result showed that the expression of *ATG32* was increased by GPS at concentrations of 1 and 3 *μ*M (Figure 4(a), *p* < 0.01 and *p* < 0.05), respectively. We examined the effect of GPS on the replicative lifespan of the *atg32* mutant with K6001 background and chronological lifespan of the *atg32* mutant with YOM36 background. The replicative lifespan of the *atg32* mutant with K6001 background is shown in Figure 4(b). The average replicative lifespan of the K6001 strain was 7.65 ± 0.59 generations in the negative control, and the generations increased to 9.95 ± 0.51 (*p* < 0.001) and 9.95 ± 0.59 (*p* < 0.001) after treatment of Res at 10 *μ*M and GPS at 1 *μ*M, respectively. The replicative lifespan of the *atg32* mutant of K6001 was 7.18 ± 0.53 generations in the control, and the generations were 7.45 ± 0.49 and 6.30 ± 0.37 after treatment with Res at 10 *μ*M and GPS at 1 *μ*M, respectively, suggesting that Res and GPS cannot extend the replicative lifespan of the *atg32* mutant of K6001. These results implied that *ATG32* was involved in the effect of extending the replicative lifespan of GPS. The chronological lifespan assay was performed using YOM36 wild type and *atg32* mutant with YOM36 background yeast. The absence of *ATG32* gene shortened the chronological lifespan of yeast (Figure 4(c)). A significant change was found on the survival rate of the *atg32* mutant with YOM36 background after treatment with GPS at 1 *μ*M (Figure 4(c)), suggesting that *ATG32* was not involved in the effect of extending the chronological lifespan of GPS. These results indicated that GPS extended the replicative lifespan through the regulation of mitophagy that requires *ATG32*.

### 3.4. GPS Increases the Survival Rate of Yeast under Oxidative Stress Condition

**Figure 5:**
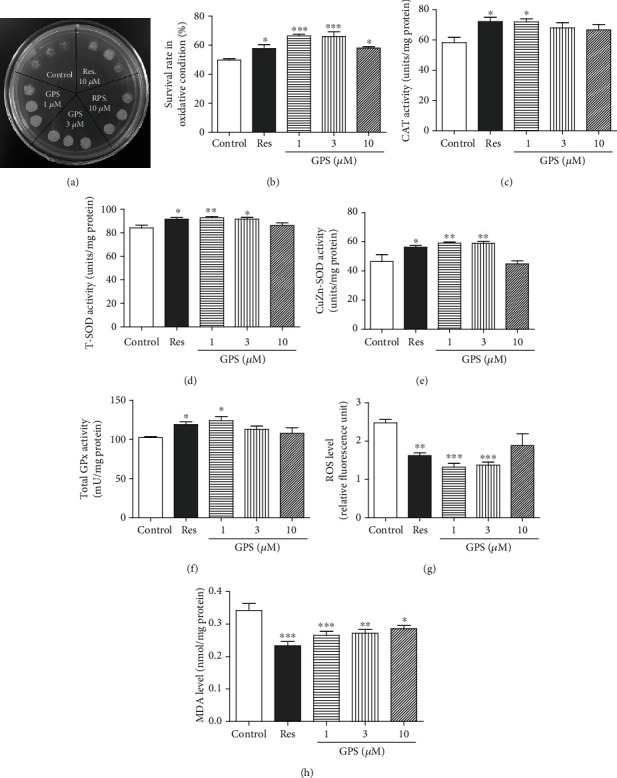
Effect of GPS on the survival rate of yeast under oxidative stress condition and antioxidative enzyme activity in yeast. (a) Photograph of yeast growth after treatment of GPS under oxidative stress condition induced by H_2_O_2_ at 10.5 mM. (b) The survival rate changes in yeast under oxidative conditions at 5 mM H_2_O_2_. (c–f) The changes in CAT, T-SOD, SOD1, and GPx enzyme activities in yeast after treatment of GPS for 48 h. (g, h) Effect of GPS on ROS and MDA levels. Each experiment is repeated thrice. ∗, ∗∗, and ∗∗∗ indicate significant differences from the corresponding control (*p* < 0.05, *p* < 0.01, and *p* < 0.001), respectively.

Oxidative stress is a major risk factor of aging and age-related diseases, and strong oxidative stress results in the damage of DNA, lipid peroxidation, and dysfunction of protein. Qualitative and quantitative experiments were performed to evaluate the antioxidative activity of GPS, as shown in Figures 5(a) and 5(b). The survival rates of yeast in oxidative stress were 48.8% ± 0.8 in the control group, 57.7% ± 2.6 in the positive control with Res-treated group (*p* < 0.05), 66.4% ± 1.1 in the GPS at 1 *μ*M treated group (*p* < 0.01), 66.0% ± 3.2 in the GPS at 3 *μ*M group (*p* < 0.01) and 58.1% ± 0.9 in the treatment of GPS at 10 *μ*M (*p* < 0.05), respectively. These results indicated that GPS has antioxidative stress and plays an important role in the antiaging activity of GPS in yeast.

### 3.5. GPS Improves the CAT, SOD, and GPx Activities in Yeast

CAT, SOD, and GPx play important roles in antioxidative stress. The enzyme activities of CAT, total-SOD, CuZn-SOD and GPx in yeast were measured after treatment with Res at 10 *μ*M and GPS at 1, 3, and 10 *μ*M to confirm the antioxidative effect of GPS. The enzyme activity (units/mg) of CAT significantly increased from 58.3 ± 3.4 to 72.2 ± 2.8, 72.0 ± 2.0, 68.0 ± 3.4, and 66.7 ± 3.7 (*p* < 0.05, *p* < 0.05, *p* < 0.05, and *p* < 0.05), respectively (Figures 5(c)). As shown in Figures 5(d) and 5(e), the enzyme activity (units/mg) of total-SOD significantly increased from 84.2 ± 2.3 to 91.6 ± 1.4, 92.8 ± 1.2, 91.7 ± 1.4, and 86.2 ± 2.1 (*p* < 0.05, *p* < 0.01, and *p* < 0.05), respectively. The enzyme activity (units/mg) of CuZn-SOD significantly increased from 46.1 ± 4.8 to 56.4 ± 1.0, 59.1 ± 0.7, 58.9 ± 1.2, and 44.8 ± 2.1 (*p* < 0.05 and *p* < 0.01 and *p* < 0.01, respectively. The enzyme activity of GPx (mU/mg) significantly increased from 102.4 ± 1.1 to 119.0 ± 3.6, 124.0 ± 5.4, 112.7 ± 4.4, and 107.7 ± 7.2 (*p* < 0.05, *p* < 0.05), respectively (Figure 5(f)). These results confirmed that GPS exhibited an antioxidative activity in yeast.

### 3.6. GPS Decreases the ROS and MDA Levels in Yeast

Increased production of ROS can cause mitochondrial deterioration and global cellular damage, thereby supporting the role of ROS in aging [22]. MDA is a product of lipid peroxidation of polyunsaturated fatty acids and is a popular indicator used to determine oxidative stress [19]. Thus, we evaluated the levels of ROS and MDA after treatment with Res at 10 *μ*M and GPS at 1, 3, and 10 *μ*M for 24 h in yeast. As shown in Figures 5(g) and 5(h), the level of ROS decreased from 2.47 ± 0.09 to 1.62 ± 0.07, 1.32 ± 0.10, 1.38 ± 0.08, and 1.88 ± 0.31 (*p* < 0.01, *p* < 0.001, *p* < 0.001, and *p* < 0.05), respectively. The level of MDA decreased from 0.34 ± 0.02 to 0.23 ± 0.01, 0.27 ± 0.01, 0.27 ± 0.01, and 0.29 ± 0.01 (*p* < 0.001, *p* < 0.001, *p* < 0.01, and *p* < 0.05), respectively. These results suggested that GPS can significantly reduce the ROS level and production of MDA.

### 3.7. SOD1, SOD2, UTH1, and SKN7 Involved in the Antiaging Effect of GPS

**Figure 6:**
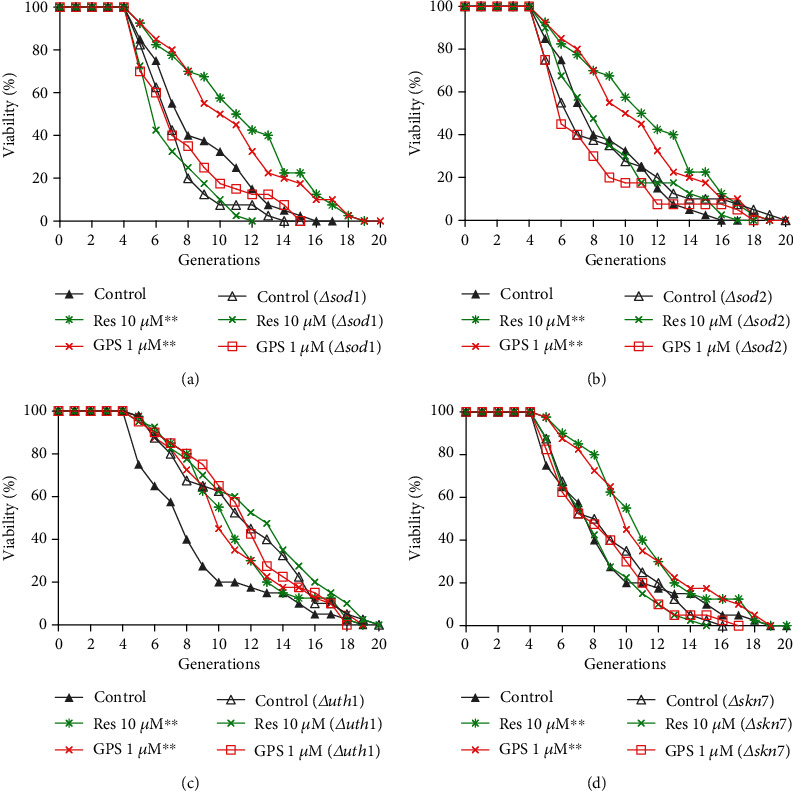
Effect of GPS on the replicative lifespans of *Δ sod1* (a), *Δsod2* (b), *Δ uth1* (c), and *Δskn7* (d) yeast with K6001 background. The procedure for the replicative lifespan assay is the same as that for the K6001 lifespan assay. Each experiment is conducted thrice. ^∗∗^ indicates significant difference between the control group of K6001 and GPS-treated group at *p* < 0.01.

*SOD1*, *SOD2*, *UTH1*, and *SKN7* are antioxidative or oxidative-related genes. Replicative lifespan of K6001 strain and *sod1*, *sod2*, *uth1*, and *skn7* mutants with K6001 background were performed to evaluate the effects of GPS on these genes. The results are demonstrated in Figures 6(a)–6(d). The average replicative lifespan of the K6001 strain was 7.80 ± 0.49 generations in the negative control, and the generations increased to 10.38 ± 0.64 (*p* < 0.01) and 9.88 ± 0.60 (*p* < 0.01) after the treatment of positive control (Res at 10 *μ*M) and GPS at 1 *μ*M, respectively (Figures 6(a) and (6b)). The average replicative lifespan of the *sod1* mutant of K6001 was 6.35 ± 0.31 generations in the negative control, and the generations were 6.33 ± 0.33 and 6.90 ± 0.49 after treatment of Res at 10 *μ*M and GPS at 1 *μ*M, respectively (Figure 6(a)), suggesting that Res and GPS did not extend the replicative lifespan of the *sod1* mutant of K6001. In the case of the negative control, the average replicative lifespan of the *sod2* mutant of K6001 was 7.60 ± 0.63 generations, and the generations were 7.95 ± 0.54 and 6.68 ± 0.51 after treatment with Res at 10 *μ*M and GPS at 1 *μ*M, respectively (Figure 6(b)), suggesting that Res and GPS did not prolong the replicative lifespan of the *sod2* mutant of K6001. The average replicative lifespan of the K6001 strain was 7.65 ± 0.59 generations in the negative control, and the generations increased to 9.95 ± 0.51 (*p* < 0.01) and 9.95 ± 0.59 (*p* < 0.01) after treatment of Res at 10 *μ*M and GPS at 1 *μ*M, respectively (Figures 6(c) and 6(d)). The replicative lifespan of the *uth1* mutant of K6001 was 10.75 ± 0.65 generations in the negative control, and the generations were 11.50 ± 0.68 and 10.83 ± 0.58 after treatment with Res at 10 *μ*M and GPS at 1 *μ*M, respectively (Figure 6(c)), suggesting that Res and GPS did not extend the replicative lifespan of the *uth1* mutant of K6001. The replicative lifespan of the *skn7* mutant of K6001 was 7.98 ± 0.52 generations in the negative control, and the generations were 7.33 ± 0.43 and 7.58 ± 0.49 after treatment with Res at 10 *μ*M and GPS at 1 *μ*M, respectively (Figure 6(d)), suggesting that Res and GPS did not extend the replicative lifespan of the *skn7* mutant with the background of K6001. These results indicated that *SOD1*, *SOD2*, *UTH1*, and *SKN7* are involved in the antiaging effect of GPS.

## 4. Discussion

*G*. *rigescens* Franch is a traditional Chinese medicine produced in Yunnan and Guizhou Provinces, China. Our research group has long been engaged in the chemical and biological studies on active natural products and their derivatives from *G*. *rigescens* for drug discovery against Alzheimer's disease and antiaging. In our previous studies, many novel molecules were isolated from *G*. *rigescens* and hundreds of their derivatives were synthesised. We discovered the lead compound, tetradecyl 2,3-dihydroxybenzoate through a structure–activity relationship study. This compound demonstrated significant neuritogenic effects in cell culture and animal models [31]. These results implied that we may have discovered substances that have cognition improvement from *G*. *rigescens*, as described in *Sheng Nong's Herbal Classic*. However, the active molecules on antiaging effect described in *Sheng Nong's Herbal Classic* are still under discovery. In the present study, GPS, which is a major component in *G*. *rigescens*, was isolated as the antiaging molecule under the guidance of the yeast K6001 replicative lifespan bioassay system. A previous literature reported that GPS has antioxidant, hepatoprotect, anti-inflammation, analgesic, and antibacterial effects [32]. However, intensive studies on the antiaging activity of GPS and its mechanism of action are lacking. The extension of replicative and chronological lifespans of yeast after treatment of GPS (Figures 1(b) and 1(c)) suggested that GPS had an antiaging activity on yeast.

Subsequently, two types of classical antiaging mechanisms, namely, autophagy and antioxidative stress, were investigated to understand the antiaging mechanism of GPS. Atg8 is an important component for autophagic machinery, participates in the entire process of autophagy, and is a biomarker of autophagy in yeast [33]. In our previous study, we found that autophagy was involved in the antiaging activity of cucurbitacin B (CuB), a natural product [22]. Therefore, we firstly examined whether autophagy was involved in the antiaging effect of GPS. The increase in free GFP in YOM38-GFP-Atg8 yeast after treatment of GSP (Figure 2) confirmed that autophagy played an important role in the antiaging activity of GPS. Considering that autophagy, including mitochondrial, peroxisome, and nonselective autophagies, is categorised in selective autophagy, understanding which type of autophagy plays an important role in the antiaging effect of GPS is crucial. Therefore, we focused on mitophagy, especially Atg8 and Atg32, which are essential for mitophagy [34], to perform investigation using Mito-Tracker Red CMXRos stain method, western blot analysis, lifespan assay of the *atg32* mutant yeast with K6001 or YOM36 background, and RT-PCR. The increase in free GFP and ubiquitin in the mitochondria and gene expression of *ATG32* and no changes in the replicative lifespan of the *atg32* mutant with K6001 background after treatment of GPS (Figures 3–4) clarified that mitophagy was involved in the antiaging effect of GPS. GPS, a secoiridoid glycoside, and CuB, a highly oxidised triterpenoid, belonged to completely different types of chemical molecules. They exhibited significant effect on inducing autophagy. Moreover, a phenolic type molecule, bis(4-hydroxybenzyl)ether mono-*β*-L-galactopyranoside from the *Gastrodia elata* [2], as well as GPS and CuB, showed a significant antiaging activity on yeasts. All of these three different structural types of molecules exhibited antiaging activities through antioxidative stress. The decrease of ROS and MDA levels significantly were common features of these three different structural types of compounds. Therefore, studying the similarities and differences among the three different types of molecules in inducing autophagy and antioxidative stress in the future is extremely valuable.

We investigated the effects of antioxidative stress on the antiaging effect of GPS. The increase in the survival rate under oxidative stress; enhancement of CAT, SOD, and GPx activities; and the decrease in ROS and MDA levels on yeast (Figure 5) suggested that GPS exhibited antiaging effect on yeast with the increase in antioxidant enzyme activity and antioxidative stress. We performed the replicative lifespan assay of *sod1*, *sod2*, *uth1*, and *skn7* yeast mutants with a K6001 background to provide many evidence for supporting the conclusion. The replicative lifespan assay results of yeast mutants in Figure 6 showed that *SOD1*, *SOD2*, *UTH1*, and *SKN7* were required in the antiaging effect of GPS.

In the present study, we found that SD and SC mediums had different effects on the chronological lifespan of YOM36 yeast. The survival rate of yeast in the SC medium was better than that in the SD medium (Figures 1(c) and S1(b)). The culture medium for chronological lifespan assay will be optimised in the future study.

## 5. Conclusion

The findings revealed that GPS isolated from *G*. *rigescens*, a traditional Chinese medicine, has a significant antiaging activity on yeast. GPS prolonged the replicative and chronological lifespans through the regulation of mitophagy and antioxidative stress. GPS is an extremely high-content molecule in *G*. *rigescens* and is a representative component of the plant. This condition indicates that large quantities of the compound can be readily obtained for subsequent chemical and biological studies. This study provides an important foundation for future studies on the mechanism of GPS and evaluation of the antiaging effect of GPS in different antiaging animal models for novel drug development.

## Data Availability

The data used to support the findings of this study are included within the article.

## Supplementary Materials

Supplementary materials Figure S1: effects of GPS on replicative lifespan of K6001 and chronological lifespan of YOM36 in SD medium. Figure S2: The origin data of western blotting analysis of total lysates in Figure 2(c) and mitochondrial fraction in Figures 3(c) and (d).
