# Supplementary material for: Gentiopicroside, a Secoiridoid Glycoside from Gentiana rigescens Franch, Extends the Lifespan of Yeast via Inducing Mitophagy and Antioxidative Stress
Source: Oxid Med Cell Longev. 2020 Aug 2;2020:9125752. doi: 10.1155/2020/9125752 (PMC7421792; doi:10.1155/2020/9125752)
Supplement: Supplementary materials — Figure S1: effects of GPS on replicative lifespan of K6001 and chronological lifespan of YOM36 in SD medium. Figure S2: The origin data of western blotting analysis of total lysates in Figure 2(c) and mitochondrial fraction in Figures 3(c) and (d). [file 9125752.f1.docx]

**Supplementary Materials**

**Figures**


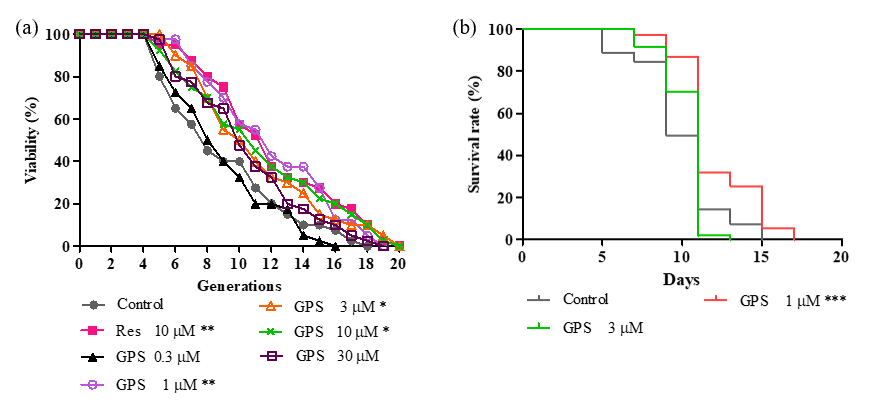


**Figure S1.** The replicative lifespan of K6001 (a) and chronological lifespan of YOM36 (b) in SC medium.


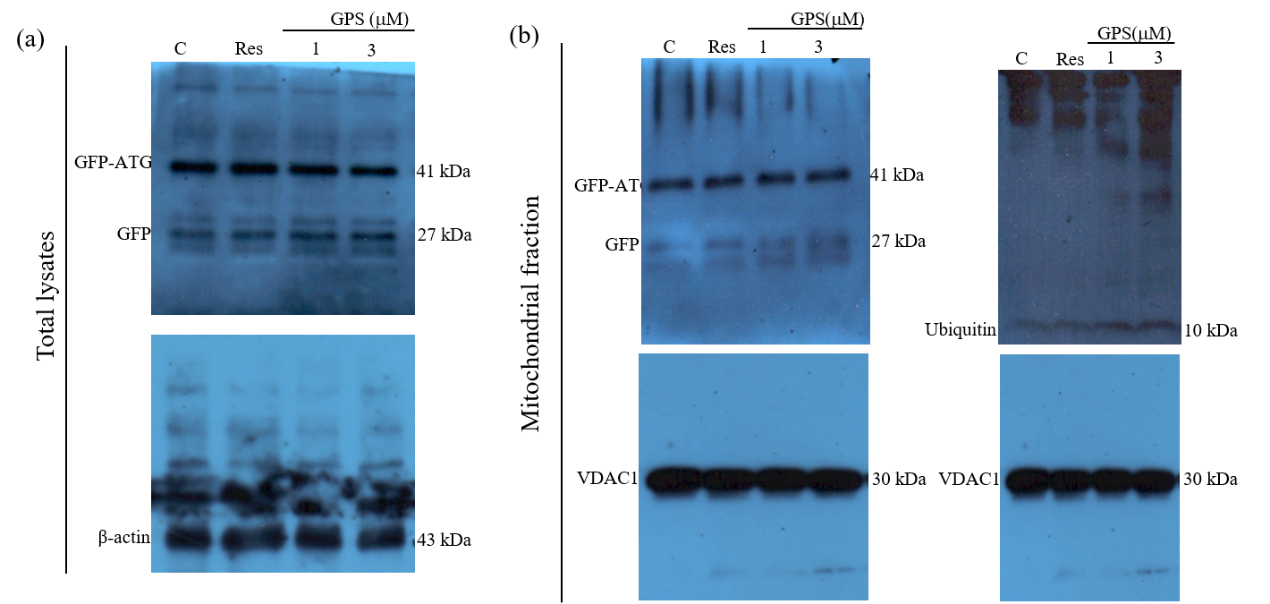


**Figure S2.** The origin data of western blotting analysis of total lysates in Figure 2 (c) and mitochondrial fraction in Figure 3 (c) and (d)
